# Role of Intravenous Iron Sucrose in Severe Anemia in Late Pregnancy: A Case Report From Rural Ballabgarh, Haryana

**DOI:** 10.7759/cureus.35472

**Published:** 2023-02-25

**Authors:** Archismita Santra, K Aparna Sharma, Neha Singh, Kapil Yadav, Shashi Kant

**Affiliations:** 1 Community Medicine, All India Institute of Medical Sciences, Raipur, Raipur, IND; 2 Centre for Community Medicine, All India Institute of Medical Sciences, New Delhi, New Delhi, IND; 3 Obstetrics and Gynaecology, All India Institute of Medical Sciences, New Delhi, New Delhi, IND

**Keywords:** parenteral iron, intravenous iron, blood transfusion, severe anemia, anemia, pregnancy

## Abstract

Severe anemia is a high-risk factor in pregnancy and needs to be treated appropriately to prevent poor maternal and fetal outcomes. A pregnant woman with severe anemia reluctant for blood transfusion due to issues of accessibility was given four doses of 300 mg intravenous iron sucrose (IVIS) in 300 ml normal saline starting at 31 weeks 5 days of gestation and her hemoglobin level increased by 4.2 gm/dl over a period of five weeks without any complications and without any intake of iron and folic acid tablets during the entire duration. Intravenous iron sucrose is a useful intervention for severe anemia of pregnancy even in late pregnancy with rapid increase in haemoglobin levels and can be used regularly for treating severe anemia in pregnant women alternative to blood transfusion who have limited accessibility to blood transfusion facilities.

## Introduction

Iron deficiency anemia (IDA) is the most common nutritional deficiency. In pregnancy, hemoglobin level less than 11 gm/dl is considered to be anemia and severe anemia is defined as hemoglobin level less than 7 gm/dl [1]. Severe anemia is a high-risk factor in pregnancy and is found to be associated with frequent preterm birth and low birth weight as well as increased risk of maternal death [2]. Severe anemia can however be corrected with necessary interventions at the appropriate time.

In India, more than 50% of pregnant women suffer from any grade of anemia and in Haryana, 56.4% of pregnant women are anemic of whom 29.1% have moderate anemia and 2.2% have severe anemia according to National Family Health Survey (NFHS-5) [3]. Though intravenous iron infusion has been recommended for the treatment of severe anemia in pregnancy with hemoglobin levels from 5.0 gm/dl to 6.9 gm/dl up to 34 weeks of gestation, there is dilemma regarding implementation of standard of care across the country and blood transfusion remains a preferred method of treatment of severe anemia in late pregnancy as it causes rapid improvement in hemoglobin levels in limited time available [4]. In resource-poor settings, there is a lack of blood transfusion facilities and the existing facilities have lower accessibility. Therefore, intravenous iron infusion which is comparatively easily available at accessible facilities like primary health centres (PHCs), can be used as an intervention to treat severe anemia in pregnancy which can also result in rapid improvement in hemoglobin levels such that poor pregnancy outcomes are prevented. Therefore, the objective in this case study was to monitor the increase in hemoglobin levels in a pregnant woman suffering from severe anemia and non-compliant to oral iron in late pregnancy with the help of intravenous iron sucrose infusion.

## Case presentation

In this case, a pregnant woman who was diagnosed with severe anemia in the late second trimester, non-compliant to oral iron was administered intravenous iron sucrose (IVIS) from 31 weeks 5 days of gestation and the increase in her hemoglobin level was monitored over a period of five weeks.

A 28-year-old G4P3L3A0 was registered at 11 weeks of gestation under the nearest subcentre by the Accredited Social Health Activist (ASHA). She belonged to middle-class socioeconomic status and was a vegetarian, her diet mostly consisted of cereals, milk, tea and packaged foods. The diversity in her diet was limited with minimal consumption of iron-rich food items and a predominantly milk-rich diet. Her hemoglobin levels were not assessed during the registration of pregnancy as the subcentres were closed due to the COVID-19 lockdown.

The first two pregnancies for this woman were uneventful throughout the antenatal period and normal vaginal delivery was conducted at home for both these pregnancies. There was adequate spacing between the first three pregnancies. She was diagnosed with severe anemia in the third pregnancy at term for which she was given a blood transfusion. She delivered a 2.9 kg male baby at 37 weeks of gestation following the blood transfusion at the district hospital by vaginal delivery. Her third and youngest child was 2 years 11 months old male and was moderately anemic with hemoglobin of 10.5 gm/dl.

During the current pregnancy, on examination of lower palpebral conjunctiva at the second antenatal check-up at 27 weeks 1 day of gestation, she was found to have severe pallor and hemoglobin level measured using capillary blood by Hemocue Hb 301 (Hemocue, Ängelholm, Sweden) was 6.3 gm/dl. While measuring hemoglobin level from capillary blood, the first drop of blood was wiped off with sterile cotton before taking the blood sample. On diagnosis of severe anemia, she was advised blood transfusion for which she did not comply with as she needed to go to the district hospital for the same. Also, non-availability of donor was a limiting factor for blood transfusion. She experienced the same challenge during her last pregnancy at term following the advice of a blood transfusion at the district hospital. Moreover, during the last gestation, she experienced labour pain and delivered on the same day of getting transfused at the hospital for which she was not prepared. She was also reluctant to go to the nearest PHC for parenteral iron infusion which was 3 km away from her residence as she did not have personal means of transport. Thus, she was advised IFA tablets twice daily until the next follow-up visit. However, she reported that she was provided with iron and folic acid (IFA) tablets previously at 12 weeks of gestation by the ASHA but consumed them irregularly. Therefore, she was counselled on the proper ways of consumption of IFA and was advised to limit her tea intake and encouraged to consume iron-rich foods like pulses, jaggery, citrus fruits and green leafy vegetables.

During the next follow-up antenatal check-up at 31 weeks 5 days, her hemoglobin level was 5.1 gm/dl and she reported poor compliance to oral iron and folic acid (IFA) tablets due to side effects in the form of epigastric discomfort and vomiting. She reported to have consumed IFA tablets for three weeks irregularly an hour after having a meal but stopped taking IFA abruptly by herself. Thus, she was started on IVIS (Venofer 20 mg iron/ml) as she gave consent for parenteral iron infusion after understanding the consequences of severe anemia in pregnancy as her hemoglobin level was declining serially. Written informed consent was obtained from her that the information collected will be kept confidential and may be used for completely academic or research purposes. According to Ganzoni’s formula, with a pre-pregnancy body weight of 50 kg and 500 mg elemental iron for replenishing the stores, target Hb of 11 gm/dl the required amount of IVIS was calculated to be 1208 mg [5]. Four doses, each of 300 mg IVIS in 300 ml of normal saline, were administered at an interval of three days. The last dose of IVIS was given at 33 weeks 2 days of gestation. No adverse reactions were noted during or after IVIS infusion. She was advised to stop consuming IFA tablets during the time span of IVIS infusion.

**Figure 1:**
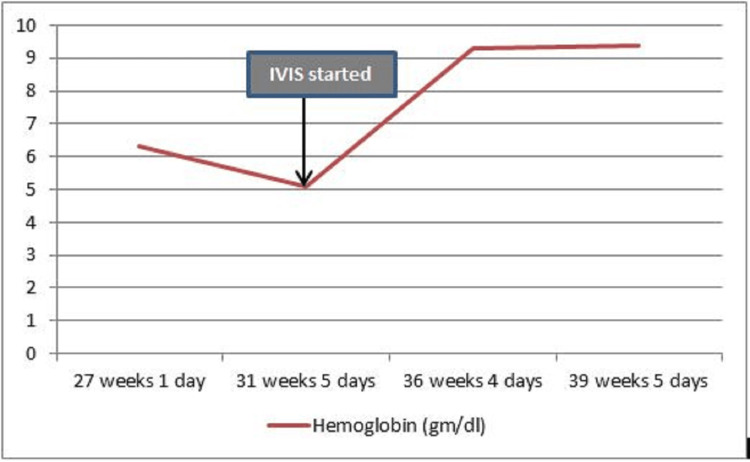
Variation in hemoglobin level with period of gestation IVIS: Intravenous iron sucrose

Her hemoglobin again measured at 36 weeks 4 days of gestation was 9.3 gm/dl. At 39 weeks 5 days of gestation, her hemoglobin was measured to be 9.4 gm/dl though she did not consume any IFA tablets throughout the period after infusion of IVIS until she delivered the baby due to previously stated epigastric discomfort. Thus, her hemoglobin level increased by 4.2 gm/dl over a period of five weeks from the initiation of IVIS with no reported adverse effects (Figure 1). The outcome of the pregnancy was a healthy male baby with a birth weight of 3.5 kg born from a vaginal delivery delivered at the nearest PHC.

## Discussion

In this case, a rapid improvement of hemoglobin level by 4.2 gm/dl points was noticed within a period of five weeks without any adverse reactions after administration of four doses of IVIS infusion after 31 weeks 5 days of gestation. As per a recent meta-analysis, it is estimated that maternal anemia contributes to 18% of perinatal mortality and 20% of maternal mortality in South Asian countries including India [6]. Anemia in the first or second trimester significantly increases the risk of low birth weight and preterm birth [6]. Prenatal iron supplementation increases birth weight and significantly reduces the risk of low birth weight [7].

According to Anemia Mukt Bharat guidelines, 2019 intravenous iron can be administered to pregnant women with severe anemia till 34 weeks of gestation [4]. The Indian Public Health Standards (IPHS) and quality benchmarks for secondary care hospitals prescribe injectable iron treatment for moderate anemia and blood transfusion is advocated for severe anemia [8,9]. The reason stated for preferring blood transfusion in late pregnancy is the limited time available till full term gestation to raise the hemoglobin to the desired level [10]. The treatment modality of blood transfusion for severe anemia in late pregnancy is sub-optimally utilised in India due to a demand and supply mismatch for blood products along with limited accessibility of transfusion centres.

Intravenous iron has fewer gastrointestinal side effects and combines the advantages of complete bioavailability with faster recovery of hemoglobin levels than oral iron. However, one of the disadvantages of IVIS infusion was the requirement of multiple infusions. This has been circumvented by the use of newer preparations like iron isomaltoside and iron carboxymaltose which allow larger infusion doses of elemental iron to be administered over a short period of time and thus obliviate multiple infusions [11]. Also in case of IVIS infusion, there is a need for trained specialists for the management of any anaphylaxis that might occur, even though IVIS has a much lower risk of anaphylactic reactions when compared to other formulations of parenteral iron formulations like iron dextran, iron gluconate or ferumoxytol [12].

In a study conducted in rural North India among pregnant women, it was found that women with severe anemia had a higher mean increase in hemoglobin than those with moderate anemia after administration of a standard dose of IVIS [13]. A randomized controlled trial comparing the safety and effectiveness of intravenous iron sucrose with standard oral iron therapy among pregnant women with moderate to severe anemia concluded that no serious adverse effects were related to IVIS which caused more increase in serum ferritin levels than oral iron [14]. In this trial, IVIS was not found to have improved maternal and fetal outcomes more than oral iron but it was reiterated that IVIS is a safer alternative to treat anemia in pregnant women when they are not compliant or intolerant to oral iron [14]. Another study conducted in North India also showed that there was no evidence of increased oxidative stress following IVIS administration [15].

## Conclusions

Thus, it can be concluded that in a resource-limited setting where there are availability/accessibility issues limiting the use of blood transfusion, IVIS can be considered as a safe alternative to blood transfusion for treating severe anemia even in late pregnancy.

## References

[1] WHO WHO (2011). Haemoglobin Concentrations for the Diagnosis of Anaemia and Assessment of Severity. Vitamin and Mineral Nutrition Information System. (WHO/NMH/NHD/MNM/11.1). https://apps.who.int/iris/bitstream/handle/10665/85839/WHO_NMH_NHD_MNM_11.1_eng.pdf.

[2] Geelhoed D, Agadzi F, Visser L (2006). Maternal and fetal outcome after severe anemia in pregnancy in rural Ghana. Acta Obstet Gynecol Scand.

[3] International Institute for Population Sciences (IIPS) and ICF (2023). National Family Health Survey (NFHS-5), India, 2019-21: Haryana. http://rchiips.org/nfhs/NFHS-5Reports/Haryana.pdf.

[4] Reproductive and Child Health Division, Ministry of Health and Family Welfare (2019). Anemia Mukt Bharat Training Toolkit. https://anemiamuktbharat.info/wp-content/uploads/2019/11/English_Anemia-Mukt-Bharat-Training-Modules-Folder_Lowress.pdf.

[5] (2023). Total Iron Deficit calculator (Ganzoni equation). http://www.al-nasir.com/www/PharmCalc/exec_calc.php?ID=ganzoni.

[6] Rahman MM, Abe SK, Rahman MS (2016). Maternal anemia and risk of adverse birth and health outcomes in low- and middle-income countries: systematic review and meta-analysis. Am J Clin Nutr.

[7] WHO WHO (2017). Nutritional Anaemias: Tools for Effective Prevention and Control. https://www.who.int/publications-detail-redirect/9789241513067.

[8] National Health Mission, Ministry of Health and Family Welfare, Government of India (2014). Assessor's Guidebooks for Quality Assurance in Community Health Centres (First Referral Unit). http://qi.nhsrcindia.org/assessor-guidebooks-quality-assurance-community-health-centres-first-refferal-unit-0.

[9] National Health Mission, Ministry of Health and Family Welfare, Government of India (2013). Assessor's Guidebook for Quality Assurance in District Hospitals - Volume 1. https://www.kayakalpraj.org/assets/Guideline/Assessor_guidebook_for_quality_assurance_in_District_Hospitals-Volume_1.pdf.

[10] Royal College of Obstetricians and Gynaecologists (2015). Blood Transfusion in Obstetrics. Green-top Guideline No. 47. May.

[11] Achebe MM, Gafter-Gvili A (2017). How I treat anemia in pregnancy: iron, cobalamin, and folate. Blood.

[12] Wang C, Graham DJ, Kane RC (2015). Comparative risk of anaphylactic reactions associated with intravenous iron products. JAMA.

[13] Haldar P, Kant S, Yadav V (2018). Effect of intravenous iron sucrose on hemoglobin level, when administered in a standard-dose, to anemic pregnant women in rural Northern India. J Family Med Prim Care.

[14] Neogi SB, Devasenapathy N, Singh R (2019). Safety and effectiveness of intravenous iron sucrose versus standard oral iron therapy in pregnant women with moderate-to-severe anaemia in India: a multicentre, open-label, phase 3, randomised, controlled trial. Lancet Global Health.

[15] Jacob OM, Kant S, Haldar P, Kaur R, Dadhwal V, Prakash S (2020). Intravenous Iron sucrose and change in hemoglobin, ferritin, and oxidative stress markers among moderately anemic pregnant women attending a secondary care level Hospital in Northern India. Indian J Public Health.

